# Frailty among older adults in Germany: regional variation across NAKO study centers

**DOI:** 10.1186/s12939-026-02879-y

**Published:** 2026-06-03

**Authors:** Maximilian König, Stefan Rach, Rafael Mikolajczyk, Ben Schöttker, Ute Mons, Manuel Amthor, Henry Völzke, Claudia Meinke-Franze, Volker Harth, Nadia Obi, Matthias B. Schulze, Barbara Thorand, Karin Halina Greiser, Michael Leitzmann, Anne Herrmann, Wolfgang Lieb, Jasmin Kiekert, Thomas Keil, Lilian Krist, Börge Schmidt, Jana-Kristin Heise, Katharina Nimptsch, Tobias Pischon, Till Ittermann

**Affiliations:** 1 Department of Internal Medicine D – Geriatrics, Universitätsmedizin Greifswald, Greifswald, Germany; 2 Housing and Digitalization Competence Center Mecklenburg-Vorpommern, Greifswald, Germany; 3 Department of Epidemiological Methods and Etiological Research, Leibniz Institute for Prevention Research and Epidemiology – BIPS, Bremen, Germany; 4 Institute for Medical Epidemiology, Biometrics, and Informatics, Interdisciplinary Center for Health Sciences, Medical Faculty of the Martin Luther University Halle-Wittenberg, Halle (Saale), Germany; 5 Division of Clinical Epidemiology and Aging Research, German Cancer Research Center (DKFZ), Heidelberg, Germany; 6 Network Aging Research, University of Heidelberg, Heidelberg, Germany; 7 Division of Primary Cancer Prevention, German Cancer Research Center (DKFZ), Heidelberg, Germany; 8 Medical Faculty Mannheim, Heidelberg University, Mannheim, Germany; 9 Department of Study of Health in Pomerania/Clinical-Epidemiological Research, Institute for Community Medicine, Universitätsmedizin Greifswald, Greifswald, Germany; 10 Institute for Occupational and Maritime Medicine (ZfAM), University Medical Center Hamburg-Eppendorf, Hamburg, Germany; 11 Department of Molecular Epidemiology, German Institute of Human Nutrition Potsdam-Rehbruecke, Nuthetal, Germany; 12 Institute of Nutritional Science, University of Potsdam, Nuthetal, Germany; 13 Institute of Epidemiology, Helmholtz Zentrum München, German Research Center for Environmental Health, Neuherberg, Germany; 14 Institute for Medical Information Processing, Biometry and Epidemiology (IBE), Faculty of Medicine, LMU Munich, Pettenkofer School of Public Health, Munich, Germany; 15 Division of Cancer Epidemiology, German Cancer Research Center (DKFZ), Heidelberg, Germany; 16 Institute for Epidemiology and Preventive Medicine, University Medicine Regensburg, Regensburg, Germany; 17 Department of Hematology and Medical Oncology, University Hospital Regensburg, Regensburg, Germany; 18 Institute of Epidemiology, Christian-Albrechts-University of Kiel and University Hospital Schleswig-Holstein (UKSH), Campus Kiel, Kiel, Germany; 19 Institute for Epidemiology and Prevention, Universitätsklinikum Freiburg, Freiburg, Germany; 20 Institute of Social Medicine, Epidemiology, and Health Economics, Charité- Universitätsmedizin Berlin, Berlin, Germany; 21 Institute of Clinical Epidemiology and Biometry, University of Würzburg, Würzburg, Germany; 22 State Institute of Health I, Bavarian Health and Food Safety Authority, Erlangen, Germany; 23 Institute for Medical Informatics, Biometry and Epidemiology, University Hospital of Essen, University of Duisburg-Essen, Essen, Germany; 24 Department of Epidemiology, Helmholtz-Center for Infection Research (HZI), Braunschweig, Germany; 25 Molecular Epidemiology Research Group, Max Delbrück Center for Molecular Medicine (MDC) in the Helmholtz Association, Berlin, Germany; 26 Biobank Technology Platform, Max Delbrück Center for Molecular Medicine (MDC) in the Helmholtz Association, Berlin, Germany; 27 Charité - Universitätsmedizin Berlin, Corporate Member of Freie Universität Berlin and Humboldt-Universität zu Berlin, Berlin, Germany

**Keywords:** Frailty, Prevalence, Regional, Spatial, Older adults

## Abstract

**Background:**

Frailty is a geriatric syndrome associated with increased morbidity, disability, and mortality. While its prevalence has been studied extensively in individual cohorts and patient populations, national prevalence data are scarce and regional variations within Germany remain largely unexplored. This study examined the frequency of frailty and its regional variation across the 18 NAKO (German National Cohort) study centers using a uniform frailty index.

**Methods:**

We analysed baseline data collected between 2014 and 2019 from 39,248 participants aged 61–75 years enrolled in the NAKO study, a large population-based cohort recruited across 18 study centers in Germany. Frailty was measured using a 40-item Frailty Index (FI), with FI ≥ 0.25 indicating frailty. Regional variations in frailty were first examined using crude frequencies and subsequently analysed with logistic regression, adjusting for key confounders, including age, sex, and sociodemographic and socioeconomic factors, to account for compositional differences across centers.

**Results:**

The crude frequency of frailty in the total sample was 7.7% (95% CI: 7.5–8.0), while 34.1% were prefrail. Frailty frequencies showed a nearly twofold spread (5.4%–10.2%) across study sites, being highest in Essen, Düsseldorf, Regensburg, Saarbrücken, and Berlin, and lowest in Freiburg, Münster, and northern German centers. Higher age, lower socioeconomic status and weaker social networks were all independently associated with frailty status. However, while these compositional factors contributed to the variability observed across centers, they did not fully account for it.

**Conclusion:**

Notable regional differences in frailty are evident across NAKO study centers in Germany, even after accounting for population composition (age, sex, and socioeconomic factors). This residual heterogeneity suggests that contextual factors - such as regional healthcare access, environmental exposures, or structural policies - contribute to frailty development beyond individual risk factors.

**Supplementary Information:**

The online version contains supplementary material available at 10.1186/s12939-026-02879-y.

## Introduction

Frailty is a common geriatric syndrome in older adults, conceptualized either as the presence of three or more criteria such as diminished strength, slow walking speed, exhaustion, unintended weight loss, and low physical activity (Fried frailty phenotype, FP) or as a state of accumulated health deficits (frailty index, FI) [1–3]. A multifactorial decline driven by biological, physiological, and environmental factors across multiple systems ultimately leads to reduced physiological reserves and increased vulnerability to stressors [4–7]. Frailty is strongly associated with adverse health outcomes, including disability, hospitalization, and mortality [8–11]. Across population-based studies of community-dwelling older adults in North America, Europe, Asia, and Oceania, about 2.5–10% are estimated to be frail and 30–40% pre-frail [6, 12, 13]. Given the ongoing trend of population aging and the rising prevalence of frailty in more recent generations of older adults, the importance of frailty for public health and medical practice is expected to grow in the coming years [14].

Current evidence views frailty as a dynamic and potentially reversible process, indicating that targeted interventions may slow or prevent its progression [1, 4]. Beyond medical risk factors, socioeconomic conditions, social networks, and access to resources throughout the life course drive the health inequalities that culminate in frailty in old age [12]. However, many open questions remain, including why some individuals remain robust despite the presence of risk factors and what role spatial and contextual factors across different scales play in frailty development.

Recent European studies, using data from the Survey of Health, Ageing and Retirement in Europe (SHARE), the Nord-Trøndelag Health Study (HUNT), the Studie zur Gesundheit Erwachsener in Deutschland (DEGS1), and the English Longitudinal Study of Ageing (ELSA), have mapped frailty prevalence across multiple European countries [13, 15–17]. These studies revealed substantial variation both within and between countries, particularly between urban and rural areas, central and peripheral areas, and regions of different wealth. Yet, despite the importance of such knowledge for public health interventions and healthcare planning, little is known about the spatial patterns of frailty in Germany [18, 19]. Except for one study using the nationally-representative DEGS1 dataset [17], most German frailty estimates come from combined regional cohorts [5]. These approaches were limited by relatively small sample sizes, differences in data collection periods, sample age structures, and variations in measurement methods; moreover, they did not explicitly describe regional differences across Germany [6]. As the largest population-based study in Germany, the German National Cohort (NAKO) provides a standardized framework and comprehensive lifestyle, socioeconomic, and health data to measure frailty consistently across Germany, following the established FI-approach [1, 20–22].

Our objectives were to estimate the overall frequency of frailty in NAKO participants aged 60 years and older and to examine regional variation across the 18 NAKO study centers using both unadjusted analyses and analyses adjusted for compositional factors (e.g., sociodemographic and socioeconomic characteristics). We hypothesized that the distribution of frailty differs across German regions, reflecting variation in population composition, particularly age, income, and educational attainment, as well as contextual factors.

## Methods

### Study sample

NAKO is a large, multicenter, population-based prospective cohort study conducted in Germany; its design and methodology have been described in detail elsewhere [21]. In brief, more than 205,000 women and men (aged 19–75 years at baseline) were recruited between 2014 and 2019 through age- and sex-stratified random sampling from population registries in the catchment areas of 18 study centers, located in nearly all federal states, with the exception of Thuringia, Rhineland-Palatinate, and Hesse. The overall response was 15.6%, varying between 9% and 32% across study centers. Among participants aged 60 years and older, the average response was 20.7% [22]. Participants were required to attend the study centers for all baseline assessments, which included a self-administered questionnaire, a face-to-face interview, a wide range of biomedical examinations, and the collection of biological samples. To evaluate whether participation could be improved, pilot home visits were conducted; however, these yielded only a marginal increase in response rates and did not result in significant differences in participant characteristics [23]. For this analysis, we have used baseline data only from older adults, starting from the age of 60 years (*n* = 50.490).

### Assessment of frailty

To measure frailty, we chose the FI approach due to its flexibility in operationalization using available epidemiological data and its ability to provide a continuous, multidimensional measure of frailty. We calculated a FI using 40 items derived from the available NAKO data pool, following the standard procedure outlined by Theou et al. [20]: Initially, a set of 50 candidate items was identified by a core team of researchers, including experts in epidemiology, geriatrics, and frailty (MK, PA, TI), which had to represent health-related deficits covering multiple domains including chronic diseases, symptoms, cognition, mood, sleep, medications, physical functioning and disability, oral health, psychosocial circumstances, and self-rated health. Items were selected based on their alignment with the following established FI construction criteria [20]: We systematically screened all candidate items to ensure they were age-related, neither too rare nor too common, had fewer than 10% missing values, were not highly correlated with each other and collectively represented a broad range of organ systems. Following detailed evaluation, 10 items were excluded due to limited variability, high rates of missing data, or conceptual overlap with other items, resulting in a final FI based on 40 items. For additional details and the complete list of FI items, see Supplementary Table 1. Next, following the standard procedure [20], participants with ≥ 20% of FI items missing (i.e., fewer than 32 deficits considered) were excluded (Supplementary Fig. 1).

All candidate items were recoded so that the absence of a deficit was coded as 0 and the presence of the full deficit as 1. Items with more than two response options were assigned intermediate values as appropriate (e.g., 0, 0.25, 0.5, 0.75, 1 *or* 0, 0.33, 0.66, 1). The FI was then calculated as the sum of deficits divided by the total number of items, resulting in a continuous score ranging from 0 (no deficits) to 1 (all deficits present). Higher FI values indicate greater frailty; for example, an FI of 0.1 corresponds to relatively low frailty, while an FI above 0.4 is indicative of severe frailty. Based on Rockwood et al.’s studies, which demonstrated that an FI = 0.25 has construct and predictive validity to classify community-dwelling older adults as frail or non-frail, we categorized participants as robust (FI < 0.15), pre-frail (0.15 ≤ FI < 0.25), or frail (FI ≥ 0.25) [15, 24–27].

### Individual and study-center level variables

#### Study center

The primary unit for our regional comparison was the study center (*n* = 18).

#### East/West Germany

Study centers were classified into three regions based on historical geography: the former German Democratic Republic (East), the former Federal Republic of Germany (West), and Berlin (treated as a separate category).

#### North/South of Germany

The study centers were categorized according to conventional classification into north (Kiel, Bremen, Hamburg, Hannover, Bremen, Neubrandenburg), central (Halle, Leipzig, Berlin, Essen, Düsseldorf, Münster) and south (Augsburg, Regensburg, Saarbrücken, Freiburg, Mannheim) [28].

#### Urbanization (aggregate measure at the study-center level)

The categorization of study centers by degree of urbanization followed Wolf et al. [29], who used Eurostat data to classify European local administrative units (municipalities) into three categories: cities (densely populated areas), towns and suburbs (intermediate density areas), and rural areas (thinly populated areas). The study regions ranged from highly urbanized areas (Berlin, Hamburg) with up to 4,123 inhabitants/km^2^ to rural regions (Neubrandenburg in Mecklenburg-Vorpommern) with only 732 inhabitants/km^2^.

#### Immigrant background

Immigration status was determined from individual-level data collected in the NAKO, and defined based on self-reported country of birth and parental immigration background [30].

#### Socio-economic status (SES)

SES indicators included individual-level educational attainment, and net household income.

- *Educational attainment* was assessed using the International Standard Classification of Education 1997 (ISCED-97), which distinguishes six levels ranging from primary to tertiary education.
- *Income* data was also available in the NAKO as self-reported monthly household income and mean net equivalence income was calculated using the OECD-modified equivalence scale, which adjusts household income by household size and composition.

#### Social network index

The index comprises marital/partnership status, social contacts, and integration into social groups; measured on an ordinal scale from I (isolated) - IV, with higher scores indicating greater social connectedness.

#### Self-rated health (SRH)

SRH was assessed with the question: “How would you rate your overall health?” Responses were measured on a 5-point Likert scale ranging from excellent to poor.

### Analysis and statistics

All analyses were performed using Stata SE 18.0 (StataCorp, College Station, TX).

FI mean and median values, as well as the frequency of frailty and prefrailty were calculated for the total cohort and stratified by sex, age group (5-year strata), and study site.

We investigated potential non-linear associations between age and the frequency of frailty by testing fractional polynomials in sex-specific logistic regression models adjusted for examination center using the ***mfp*** command in Stata, with the continuous age variable as exposure and ignoring complex survey design, consistent with our main analyses [31].

Logistic regression analyses were conducted to examine regional variation in frailty across study centers adjusted for age, sex, education, income, immigration background, and social net; and to investigate individual-level and regional-level contextual predictors of frailty. Covariables were identified a priori based on literature review. Missing data were low across all variables, and multivariable analyses were conducted using complete-case data. All nested models were based on the same set of participants with complete data.

To obtain adjusted frequency estimates of frailty for each study center, we used the *margins* command in Stata. This post-estimation procedure calculates predicted probabilities from the regression model, averaging over the observed distribution of covariates in the sample. Thus, the resulting frequency estimates for each study center were adjusted for the covariates included in the model, providing standardized estimates that are comparable across centers.

All analyses, including descriptive statistics, group comparisons, and logistic regression models, were conducted at the individual level using conventional methods without survey commands. Between-center variance was minimal (ICC ≈ 0.006, calculated from a random-intercept model), indicating that only a small proportion of the total variance in frailty frequency was attributable to differences between study centers. Although large cluster sizes could theoretically inflate standard errors, the very low ICC and small number of clusters (18 study centers) made cluster-robust standard errors unstable. Therefore, conventional standard errors were used throughout, with negligible impact on point estimates, confidence intervals, and inference. Similarly, correction weights were not applied in the main analyses. However, sensitivity analyses were conducted using design weights, with results presented in Supplementary Tables 4 and 5. Multicollinearity was assessed using Variance Inflation Factors (VIF).

## Results

### Sample characteristics

Of 50,490 NAKO participants aged ≥ 60 years, 11,242 (22.3%) were excluded due to ≥ 20% missing FI items (i.e., fewer than 32 of 40 deficits available; Supplementary Table 1), leaving 39,248 participants for analysis (Supplementary Figs. 1 and 6). Most participants were aged 61–71 years, with only 250 individuals in the 72–75 years age group. The sex distribution was approximately balanced, with 18,742 females (47.8%). Further basic characteristics of the sample are presented in Table 1.

### Characteristics of the frailty index

**Fig. 1:**
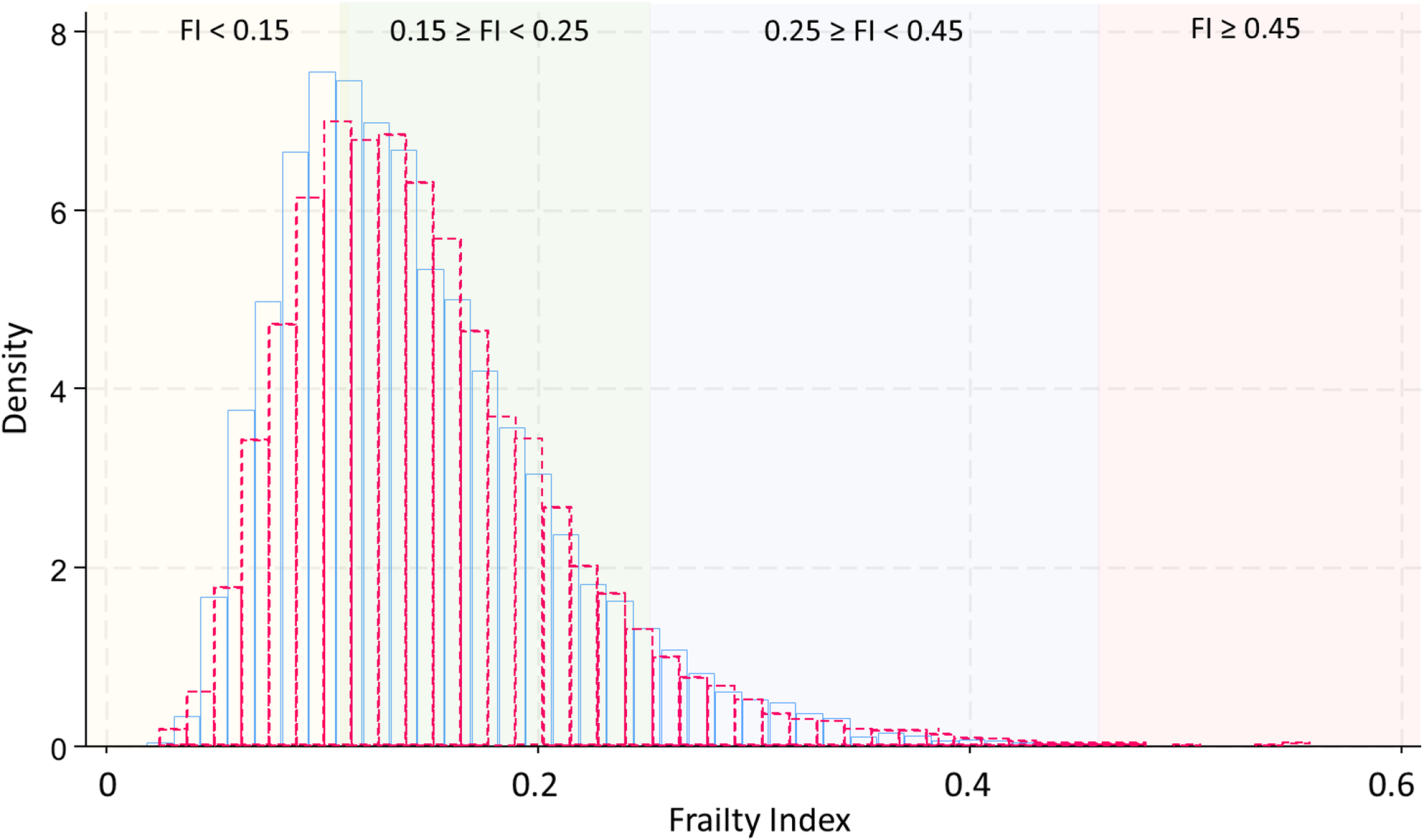
Distribution of the frailty index (FI) by sex in older participants of the NAKO study (age range: 61–75 years, *n* = 39,248). The blue histogram represents men, and the red outline represents women. The y-axis shows probability density (normalized such that the total area equals 1), and the FI is shown on the x-axis

The FI followed the typical right-skewed distribution reported in the literature (Fig. 1) [32], with a median of 0.14 and the 1st, 25th, 75th, 90th, and 99th percentiles equal to 0.05, 0.10, 0.18, 0.24, and 0.36, respectively. Mean FI scores were higher in females (0.16 ± 0.07) than in males (0.15 ± 0.07; *p* < 0.001), and the FI tended to increase with age (Table 1).

**Table 1:** Participants’ characteristics according to age group (*n* = 39,248)

	61–64 years (*N* = 17,547)	65–69 years (*N* = 18,664)	70–75 years (*N* = 3,037)
Age, years	62.5 ± 1.1	66.8 ± 1.4	70.5 ± 0.7
Sex, female	8,686 (49.5)	8,737 (46.8)	1,319 (43.4)
Cohabiting	13,479 (76.8)	14,344 (76.9)	2,291 (75.4)
Household size, persons	2(2;2)	2(2;2)	2(2;2)
Immigrant background	1,943 (11.1)	2,125 (11.4)	575 (18.9)
Education (ISCED97)*	5 (3; 5)	5 (3; 5)	5 (3;5)
Household income**, €	2,400 ± 1,584	2236 ± 1481	2188 ± 1490
Self-rated health	2 (2;2)	2 (2;2)	2 (2;2)
Excellent (1)	320 (1.8)	365(2.0)	62 (2.1)
Very good (2)	3,365 (19.2)	3,443 (18.5)	503 (16.6)
Good (3)	11,189 (63.9)	12,304 (66.7)	2,026 (66.9)
Fair (4)	2,458 (14.0)	2,375 (12.8)	420 (13.9)
Poor (5)	174 (1.0)	139 (0.8)	19 (0.6)
Frailty Index	0.13 (0.10; 0.18)	0.14 (0.10; 0.19)	0.15 (0.11; 0.20)

Notes and abbreviations: Data are presented as mean ± SD, numbers(percentages), or median (25th; 75th percentile), *ISCED97 levels: 0 = Early childhood education, 1 = Primary education, 2 = Lower secondary education, 3 = Upper secondary education, 4 = Post-secondary non-tertiary education, 5 = First stage of tertiary education, 6 = Second stage of tertiary education (ordinal), ** = net equivalence income

### Frequency of frailty

**Fig. 2:**
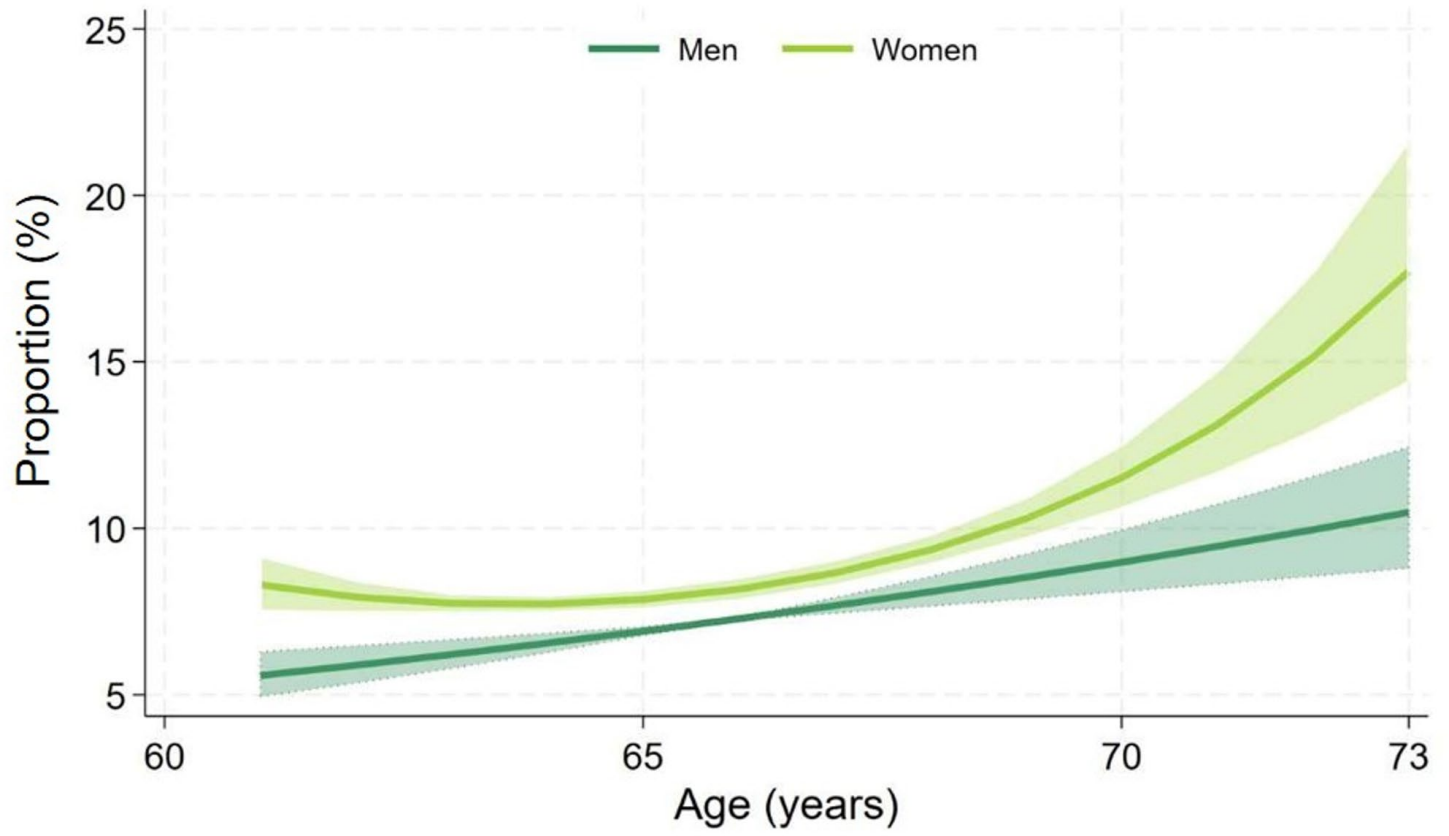
Sex-specific logistic regression models (adjusted for study site) using fractional polynomials testing for non-linear associations between age and frailty proportion. Shaded areas represent 95%-confidence intervals. Frailty increased linearly with age in men and showed an accelerated non-linear increase with age in women. The curves were cut at age 73 due to generally few participants aged 74–75, particularly there were no women aged 74 or 75

Frailty (FI ≥ 0.25) and prefrailty (0.15 ≤ FI < 0.25) were recorded in 7.7% (95% CI 7.5–8.0%) and 34.1% (95% CI 33.6–34.6%) of the sample, respectively. The frequency of both increased with age (Table 2 and Fig. 2), and was higher in women than in men: frailty affected 8.2% of women (95% CI 7.9–8.7%) compared to 7.2% of men (95% CI 6.9–7.6%, *p* < 0.001), and prefrailty 37.1% of women (95% CI 36.3–37.8%), versus 31.3% of men (95% CI 30.7–32.0%).

**Table 2:** Frailty status by sex and age group in the NAKO

	61–64 years			65–69 years			70–75 years		
	Men	Women	Total	Men	Women	Total	Men	Women	Total
Robust, %	64.9 (63.9–65.9)	58.7 (57.6–59.7)	61.8 (61.1–62.6)	60.0 (59.0–60.9)	52.5 (51.4–53.5)	56.5 (55.7–57.2)	51.7 (49.3–54.0)	42.8 (40.1–44.4)	47.8 (46.0–49.6)
Prefrail, %	28.9 (28.0–29.9)	33.7 (32.7–34.7)	31.2 (30.6–32.0)	32.2 (31.3–33.1)	39.2 (38.2–40.2)	35.5 (34.8–36.2)	38.9 (36.6–41.2)	45.4 (42.7–48.1)	41.7 (40.0–43.5)
Frail, %	6.1 (5.6–6.6)	7.7 (7.1–8.3)	6.8 (6.5–7.2)	7.9 (7.3–8.3)	8.3 (7.7–8.9)	8.1 (7.7–8.5)	9.4 (8.1–10.9)	11.8 (10.2–13.7)	10.5 (9.4–11.6)

Notes: Values are presented as frequency (%) with corresponding 95% confidence intervals. Frailty was categorized into robust (Frailty Index [FI] < 0.15), prefrail (0.15 ≥ FI < 0.25) and frail (FI ≥ 0.25) [15, 24, 25], Absolute values for each category can be found in Supplementary Table 7

Testing for non-linear associations of age with frequency of frailty revealed a linear increase of frailty frequency with age for men and a slightly accelerated non-linear increase over age in women (Fig. 2).

### Regional variation across study centers

**Fig. 3:**
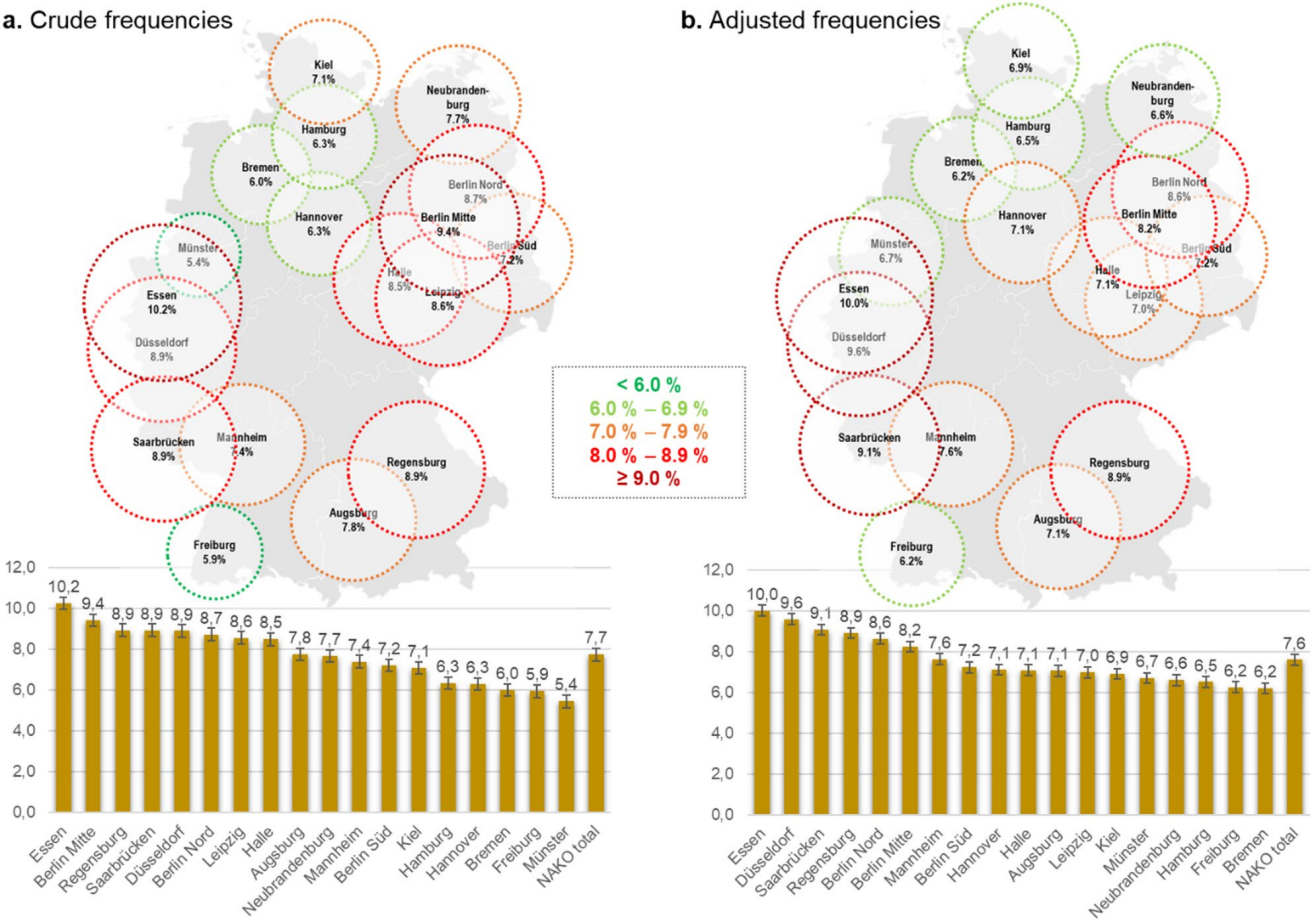
Regional variation in the frequency of frailty across NAKO study centers. (**a**) Crude frequencies representing observed proportions of frailty within each center (*n* = 39,248); (**b**) Predicted frequencies (*n* = 33,659), estimated by aggregating predicted probabilities from logistic regression models adjusted for age, sex, education, income, migration background, and social network. Error bars represent ± 1 standard error of the observed proportions in (**a**), and ± 1 standard error of the model-predicted probabilities in (**b**)

There was considerable variation in the frequency of frailty across the different study centers spanning Germany. The crude frequency of frailty ranged from 5.4% (95% CI 4.5–8.5%) in Münster to 10.2% (95% CI 9.0–11.6%) in Essen (Fig. 3a).

It was notable that the Northern German study centers and their catchment areas—Hamburg, Bremen, Kiel, Hannover, and Neubrandenburg—consistently fell in the lower half, showing a lower crude frequency of frailty compared with most other NAKO centers in Central and Southern Germany. The pooled crude frequency of frailty was 6.8%, 8.4%, and 7.8% in the northern, central, and southern study sites, respectively (χ² test, *p* < 0.001). Contrary to this general observation, Münster in North Rhine Westphalia (located in northwestern Germany) and Freiburg in the South-West (Baden-Württemberg) showed a particularly low crude frequency (5.4% and 5.9%, respectively). On the other hand, study sites in large metropolitan areas like in the Rhine-Ruhr area of North Rhine-Westphalia (Essen, Düsseldorf) or in Berlin-Mitte and Leipzig showed rather high frailty. Importantly, the ranking of study sites remained largely consistent when based on the mean FI rather than the frequency of frailty (Supplementary Table 2).

Although a difference was observed, the crude frequency of frailty between study sites in Eastern (former German Democratic Republic) and Western Germany (former West Germany) was modest (8.2% vs. 7.4%, *p* = 0.005), with Berlin completely excluded from the comparison as a special case. Furthermore, there was no evidence of relevant disparities between study centers in more urban versus more rural regions of Germany according to the established classification by Wolf et al. [29] (*p* = 0.862; Supplementary Table 3), although the study centers with the highest frequency of frailty were clearly those in large metropolitan centers (Rhine-Ruhr and Berlin Mitte and Nord).

Notably, the distribution and average of indicators of socioeconomic status, as well as sex and other social and demographic factors differed between study centers (Supplementary Table 2). For example, there were marked net equivalence income differences between former West and East Germany.

Adjusting for age and sex in a multivariable logistic regression (Model 2) analysis did not materially alter the pattern of centers with above- or below-average odds of frailty. However, after adjusting for socio-economic indicators, immigration status, and social network (Model 3), the odds of frailty (relative to Augsburg) moved closer to 1 in previously low-frailty centers (Münster, Freiburg, and northern German Centers), while they increased further in centers that already had high frailty levels (Essen, Düsseldorf, Regensburg, and Saarbrücken) (Table 3). The predicted frequencies for the individual centers remained relatively stable, as did the overall pattern—that is, whether a center exhibited relatively high or low frailty levels. (Fig. 3). Accounting for compositional differences led to a considerable shift in the rankings of Münster (down) and Neubrandenburg (up). Centers at the top of the ranking - Essen, Düsseldorf, Regensburg, Saarbrücken, Berlin Mitte, and Berlin Nord - largely retained their unfavorable positions. The findings indicated that regional variation is partly explained by differences in population composition, yet persistent disparities after accounting for compositional differences pointed to additional structural or contextual determinants.

In the multivariable model, female sex was no longer associated with frailty, whereas age, socio-economic indicators, and social network remained strong, independent predictors. Compared with participants aged 61–64 years, odds of frailty increased with age, reaching OR = 1.15 (95% CI 1.05–1.25) for ages 65–69 and OR = 1.64 (95% CI 1.41–1.90) for ages 70–75. Higher income, higher educational attainment, and a stronger social network were each independently associated with lower odds of frailty. Including urbanization as an additional covariate did not change the results; the coefficient was not statistically significant (OR 0.93, 95% CI 0.76–1.14, *p* = 0.493) and was therefore not included in the final model.

Sensitivity analyses using design weights to account for sampling design effects yielded largely consistent results (Supplementary Tables 4 and 5).

**Table 3:** Individual-level associations of frailty (multivariable logistic regression), *n* = 33,659

	Model 1	Model 2	Model 3
Male Sex	—	1(ref.)	1(ref.)
Female Sex	—	1.17 (1.08–1.27), *p* < 0.001	1.00 (0.92–1.09), *p* = 0.992
Age, years	—	1.05 (1.04–1.07), *p* < 0.001	1.04 (1.03–1.06), *p* < 0.001
Income, ordinal	—	—	0.73 (0.71–0.76), *p* < 0.001
Education, ordinal	—	—	0.87 (0.83–0.90), *p* < 0.001
No immigrant background	—	—	1(ref.)
Immigrant background	—	—	1.05 (0.92–1.19), *p* = 0.470
Social network index, ordinal	—	—	0.79 (0.74–0.83), *p* < 0.001
Augsburg	1(ref.)*	1(ref.)*	1(ref.)*
Regensburg	1.29 (1.04–1.60), *p* = 0.020	1.28 (1.30–1.59), *p* = 0.026	1.30 (1.05–1.62), *p* = 0.018
Mannheim	1.03 (0.83–1.30), *p* = 0.767	1.03 (0.82–1.29), *p* = 0.811	1.07 (0.85–1.35), *p* = 0.561
Freiburg	0.77 (0.60–0.98), *p* = 0.034	0.76 (0.60–0.98), *p* = 0.029	0.87 (0.68–1.11), *p* = 0.249
Saarbrücken	1.22 (0.97–1.54), *p* = 0.082	1.24 (0.99–1.56), *p* = 0.067	1.31 (1.03–1.65), *p* = 0.025
Essen	1.42 (1.15–1.74), *p* = 0.001	1.42 (1.16–1.74), *p* = 0.001	1.46 (1.19–1.80), *p* < 0.001
Münster	0.72 (0.57–0.93), *p* = 0.010	0.73 (0.57–0.94), *p* = 0.013	0.92 (0.71–1.18), *p* = 0.495
Düsseldorf	1.28 (1.02–1.62), *p* = 0.035	1.27 (1.01–1.60), *p* = 0.041	1.38 (1.09–1.75), *p* = 0.007
Halle	1.08 (0.86–1.35), *p* = 0.494	1.07 (0.86–1.34), *p* = 0.548	0.99 (0.79–1.25), *p* = 0.934
Leipzig	1.15 (0.94–1.41), *p* = 0.178	1.13 (0.92–1.39), *p* = 0.235	0.97 (0.78–1.19), *p* = 0.758
Berlin Nord	1.22 (0.99–1.50), *p* = 0.062	1.23 (1.00–1.51), *p* = 0.056	1.25 (1.01–1.55), *p* = 0.042
Berlin Mitte	1.25 (1.02–1.54), *p* = 0.034	1.23 (1.00–1.51), *p* = 0.050	1.18 (0.95–1.46), *p* = 0.128
Berlin Süd	0.97 (0.77–1.21), *p* = 0.782	0.92 (0.73–1.15), *p* = 0.468	1.01 (0.80–1.27), *p* = 0.944
Hannover	0.88 (0.69–1.11), *p* = 0.276	0.86 (0.68–1.10), *p* = 0.228	0.99 (0.77–1.25), *p* = 0.905
Hamburg	0.89 (0.71–1.12), *p* = 0.319	0.86 (0.69–1.08), *p* = 0.199	0.90 (0.72–1.13), *p* = 0.374
Bremen	0.77 (0.61–0.98), *p* = 0.036	0.77 (0.61–0.98), *p* = 0.032	0.86 (0.67–1.09), *p* = 0.207
Kiel	0.93 (0.73–1.18), *p* = 0.546	0.93 (0.73–1.18), *p* = 0.557	0.97 (0.76–1.23), *p* = 0.812
Neubrandenburg	0.98 (0.80–1.19), *p* = 0.802	0.98 (0.80–1.20), *p* = 0.877	0.92 (0.75–1.13), *p* = 0.436
*Pseudo R* ^*2*^	*0.00440*	*0.00770*	*0.0347*

Notes and abbreviations: *Augsburg was chosen as reference, since it was the largest NAKO center and exhibited an average crude frailty frequency. Number of participants per site: Augsburg (3,894), Regensburg (1,881), Mannheim (1,962), Freiburg (1,985), Saarbrücken (1,881), Essen (2,030), Münster (2,075), Düsseldorf (1,539), Halle (1,884), Leipzig (2,361), Berlin Nord (2,132), Berlin Mitte (2,187), Berlin Süd (1,999), Hannover (1,969), Hamburg (2,179), Bremen (2,066), Kiel (1,834), Neubrandenburg (3,390). Model 1 included only the study centers (categorical); Model 2 included the study centers, and age, and sex; Model 3 included, in addition to the study centers, the following variables: sex, age (in years), income (ordinal), education (ordinal), immigrant background (categorial), and social network index (ordinal). Model fit was assessed using Pseudo R² as indicated for each model. Variance Inflation Factors (VIF) were all below 1.2, indicating no problematic multicollinearity among the predictors

## Discussion

This analysis provides, for the first time, evidence of important regional disparities in frailty across Germany, measured by the frailty index (FI), in a large, uniformly assessed population-based cohort. Across all NAKO study centers, 7.7% of participants aged 61–75 years were classified as frail and 34.1% as prefrail. Center-specific estimates of frailty ranged from 5.4% to 10.2%, indicating notable variation across study sites.

### Comparison with prior frailty estimates in the D-A-CH region

When comparing our results with estimates reported in other studies from the D-A-CH (Germany, Austria, Switzerland) region, substantial variation in prevalence estimates becomes apparent: not only across different frailty assessment methods—such as the FI versus FP approach—but also among studies that have exclusively used the FI approach, which, if correctly applied with a sufficiently broad set of health variables, is generally considered robust and to allow for comparability across studies [6]. The available evidence on the prevalence of frailty in Germany has been recently summarized in a systematic review by Hajek et al. [6], indicating a wide range from 2.4% to 25.6%, and a pooled frailty prevalence of 13.7% (95% CI: 9.0–18.5%) among adults aged 65 and older. In interpreting our results in comparison with these findings, the relatively young mean age (65.2 years) and narrow age distribution (mostly between 61 and 71 years) of the NAKO older adult sample should be considered. Furthermore, it is important to consider that studies employing the FI approach generally report higher frailty prevalence than those using the FP approach [6, 33]. For example the ESTHER study (mean age 62.0 years) found a prevalence of frailty of 9.7% using the FI, whereas the Berlin Aging Study II (mean age 68.2 ± 3.7 years) found much lower rates of pre-frailty (28.9%) and frailty (0.9%) based on the FP [34]. When restricting the ESTHER study participants to the NAKO age range, prevalence estimates were 8.0% (ages 60–64) and 11.7% (ages 65–69). These estimates align closely with the NAKO frailty estimates, especially for the Saarland region (8.9%), where the ESTHER study was conducted [35]. The Swiss Do-Health study (mean age 74.9 ± 4.4 years) recently reported frailty and pre-frailty prevalence based on the SHARE-FI of 7.0% and 43.7% [36]. Another population-based Swiss study has likewise reported a prevalence of 7,8% with 2 or more frailty indicators present [37]. Thus, the NAKO estimates are largely consistent with the range reported from D-A-CH countries, lending support to our approach and suggesting that the underlying prevalence may fall within this range.

### Regional variation

Regarding regional disparities within Germany, participants from Essen, Düsseldorf, Regensburg, Saarbrücken, and the Berlin Nord and Mitte centers were more frequently frail, whereas participants from Freiburg, Münster, and the northern German sites showed lower crude frailty levels. While regional differences in frailty within Germany have not been previously examined, prior studies have described substantial between-country heterogeneity within Europe with increasing frailty prevalence from North to South, albeit with some exceptions [13, 38–41]. This is in line with our result that, apart from Freiburg in the southwest, study centers in northern Germany showed the lowest frailty levels. In the UK, however, a different pattern was reported, with the prevalence of prefrailty and frailty tending to be higher in coastal areas [16], which have repeatedly been described as more deprived [42], than in inland areas. Indeed, previous cross-national studies have linked higher gross-domestic product (GDP) per capita to lower frailty prevalence [13] – with high frailty and low GDP found in the eastern European countries, but also in the South (e.g. Spain and Portugal) and low frailty found in northern countries, but also Switzerland. GDP per capita reflects a country’s overall economic wealth and serves as a macro-level proxy for resources that may support healthier aging, whereas individual income more directly captures material living conditions and access to care. Our findings confirmed the previous finding that individual-level income is negatively correlated with frailty [41], which is also consistent with broader research on socioeconomic inequalities in frailty [43]. Of note, we used net equivalence income, which is particularly well suited to reflect long-term financial resources and material living conditions in later life.

The differences between study centers across Germany likely reflect a combination of compositional and regional-contextual factors. Although inter-center differences were partly accounted for by compositional differences in the local samples — including age, sex, immigration status, education, income, and social network — marked regional disparities remained, suggesting a place-based effect. Particularly the apparent advantage of centers with low frailty was attenuated when controlling for compositional differences, as odds ratios (relative to the reference Augsburg) shifted toward one, while in the centers with the highest frailty frequencies, the odds of frailty were even further increased, causing them to retain their unfavorable positions. The observation that adjustment actually increased the odds in high-frailty centers implies that these populations may face structural or environmental stressors that exacerbate frailty risk beyond the impact of individual income or education. Such factors might include regional differences in urban infrastructure, long-term exposure to industrial pollutants - particularly in the Ruhr area - or variations in the accessibility of medical care [44–46]. Interestingly, we found no association between center-level urbanization and frailty (*p* = 0.862), (Supplementary Table 3), which may reflect the limited granularity of the measure (Supplementary Table 2). To elucidate the mechanisms driving regional disparities, future research should integrate additional environmental and other contextual factors, such as area-level socioeconomic deprivation (e.g., using the German Index of Socioeconomic Deprivation), air pollution exposure (PM2.5/NO₂) [29], green space, and walkability. Whenever feasible, future analyses should employ multi-level frameworks that integrate both more granular area-level data (e.g., city districts or neighbourhoods) and individual-level data [16]. Focusing on regions or countries with particularly high frailty levels may offer a promising and resource-efficient approach to accelerate progress in frailty research.

From the health equity perspective our results show that even in a country with universal healthcare coverage, structural and environmental, as well as socioeconomic disparities manifest as unequal “physiological reserves” at the onset of older age. Consequently, our findings underscore that public health strategies targeting frailty must transcend individual-level risk management. It is imperative to identify and address the structural and environmental determinants inherent to high-burden regions.

### Strengths and limitations

A key strength of this study is its provision of directly comparable frailty and prefrailty estimates across all NAKO study centers and their respective catchment areas in Germany.

Standardized, near-simultaneous data collection between 2014 and 2019 across all centers minimizes temporal confounding. The established frailty index, with high data completeness (median of 40/40 items) and coverage of broad domains, is another strength. Additionally, the transparent methodology (Supplementary Table 1) facilitates replication.

The exclusion of 11,242 participants due to missing variables for the FI calculation did not materially alter the cohort characteristics, indicating that the analytical sample remained highly representative of the overall NAKO population aged 61–75 (Supplementary Table 6).

However, the findings may be affected by selection bias toward healthier individuals, potentially leading to an underestimation of population-level frailty prevalence: Generally, individuals with severe illness, or frailty are less likely to participate in cohort studies — specifically the NAKO, where participation required an on-site examination at the study center [21].

While participants were recruited as sex- and age-stratified random samples from the local population registries of each study center, which were deliberately distributed across Germany, however their selection was not random. Therefore, the study population cannot be considered representative of the German general population, not least because three federal states are not represented. Moreover, rural populations were systematically underrepresented (Supplementary Tables 2 and 3).

A further limitation concerns the different response rates across study centers [22]. The NAKO study group developed correction weights to account for sampling design and differential nonresponse, adjusting for age, sex, nationality, immigration status, education, and household size, as only 15.6% of invited individuals participated. These weights are intended for use with the full NAKO sample and not with subsamples, as the calibration step only aligns the marginals of the weighting variables with the population. Therefore, it cannot be assumed that distributions within intersectional strata of the weighted subsample match those of the population [22, 47]. Still, there was the possibility to use design weights, that only account for the sampling design effects. We have repeated selected analyses using these weights (Supplementary Tables 4 and 5), obtaining largely consistent results.

Moreover, since the FI relies predominantly on self-reported conditions and functional limitations, these may be subject to recall and social desirability bias.

Due to the cross-sectional design this study could not establish causality in socioeconomic-frailty relationships. Reverse causation is possible—frailty may lead to income loss, social isolation, and functional decline.

Despite adjustment for multiple individual-level factors, important center-level characteristics were not measured, including healthcare system features (e.g. geriatric service density, preventive care access), regional health policies, environmental exposures beyond those captured, occupational history, and community-level social capital.

Due to the age limit of the NAKO study our study population only included participants aged 61 to 75 years. Thus, as discussed above, we were unable to make a statement as to the totality of older adults (65–100 years), which complicates comparisons with other cohorts that cover a broader spectrum. On the other hand, this is also a strength of our study, as frailty has only rarely been examined in this young-old age group, particularly using the FI approach.

## Conclusions

This study provides the first evidence of important regional disparities in frailty frequency in Germany. Among adults aged 61–75 years, frailty and prefrailty were observed in 7.7% and 34.1% of participants, with study center-specific frailty estimates ranging from 5.4% to 10.2%. Higher frailty frequency was observed in Essen, Düsseldorf, Regensburg, Saarbrücken, and Berlin, and lower frequency in Freiburg, Münster, and most northern NAKO centers. Differences were only partly explained by age, sex, socioeconomic factors, and immigration status, suggesting that residual compositional and regional-contextual influences—such as regional deprivation, healthcare availability, or environmental exposures—may play an important role in shaping frailty risk. These findings underscore the public health relevance of frailty and highlight the need to examine environmental, place-based and contextual determinants in greater detail to guide targeted interventions.

## Supplementary Information

Below is the link to the electronic supplementary material.

Supplementary Material 1

## Data Availability

Access to and use of NAKO data and biosamples can be obtained via an electronic application portal (https://transfer.nako.de).
