# Supplementary material for: Frailty among older adults in Germany: regional variation across NAKO study centers
Source: Int J Equity Health. 2026 Jun 3;25:138. doi: 10.1186/s12939-026-02879-y (PMC13231660; doi:10.1186/s12939-026-02879-y)
Supplement: Supplementary file 1 — Supplementary Material 1 [file 12939_2026_2879_MOESM1_ESM.docx]

Supplementary Material:

Frailty among older adults in Germany: Regional variation across NAKO study centers

Maximilian König¹^,^², Stefan Rach³, Rafael Mikolajczyk⁴, Ben Schöttker⁵^,^⁶, Ute Mons⁷^,^⁸, Manuel Amthor¹, Henry Völzke⁹, Claudia Meinke-Franze⁹, Volker Harth¹⁰, Nadia Obi¹⁰, Matthias B. Schulze¹¹^,^¹², Barbara Thorand¹³^,14^, Karin Halina Greiser¹^5^, Michael Leitzmann¹^6^, , Anne Herrmann^16, 17^, Wolfgang Lieb¹^8^, Jasmin Kiekert^19^, Thomas Keil^20,21,22^, Lilian Krist^20^, Börge Schmidt^23^, Jana-Kristin Heise^24^, Katharina Nimptsch^25^ , Tobias Pischon^25,26,27^ and Till Ittermann^9^

**Affiliations**
¹ Department of Internal Medicine D – Geriatrics, Universitätsmedizin Greifswald, Greifswald, Germany
² Housing and Digitalization Competence Center Mecklenburg-Vorpommern, Greifswald, Germany³ Department of Epidemiological Methods and Etiological Research, Leibniz Institute for Prevention Research and Epidemiology – BIPS, Bremen, Germany
⁴ Institute for Medical Epidemiology, Biometrics, and Informatics, Interdisciplinary Center for Health Sciences, Medical Faculty of the Martin Luther University Halle-Wittenberg, Halle (Saale), Germany
⁵ Division of Clinical Epidemiology and Aging Research, German Cancer Research Center (DKFZ), Heidelberg, Germany
⁶ Network Aging Research, University of Heidelberg, Heidelberg, Germany
⁷ Division of Primary Cancer Prevention, German Cancer Research Center (DKFZ), Heidelberg, Germany
⁸ Medical Faculty Mannheim, Heidelberg University, Mannheim, Germany
⁹ Department of Study of Health in Pomerania/Clinical-Epidemiological Research, Institute for Community Medicine, Universitätsmedizin Greifswald, Greifswald, Germany
¹⁰ Institute for Occupational and Maritime Medicine (ZfAM), University Medical Center Hamburg-Eppendorf, Hamburg, Germany
¹¹ Department of Molecular Epidemiology, German Institute of Human Nutrition Potsdam-Rehbruecke, Nuthetal, Germany
¹² Institute of Nutritional Science, University of Potsdam, Nuthetal, Germany
¹³ Institute of Epidemiology, Helmholtz Zentrum München, German Research Center for Environmental Health, Neuherberg, Germany

^14^Institute for Medical Information Processing, Biometry and Epidemiology (IBE), Faculty of Medicine, LMU Munich; Pettenkofer School of Public Health, Munich, Germany
¹^5^ Division of Cancer Epidemiology, German Cancer Research Center (DKFZ), Heidelberg, Germany
¹^6^ Institute for Epidemiology and Preventive Medicine, University Medicine Regensburg, Regensburg, Germany

^17^Department of Hematology and Medical Oncology, University Hospital Regensburg, Germany
¹^8^ Institute of Epidemiology, Christian-Albrechts-University of Kiel and University Hospital Schleswig-Holstein (UKSH), Campus Kiel, Kiel, Germany

^19^ Institute for Epidemiology and Prevention, Universitätsklinikum Freiburg, Freiburg, Germany

^20^ Institute of Social Medicine, Epidemiology, and Health Economics, Charité-Universitätsmedizin Berlin, Berlin, Germany.

^21^ Institute of Clinical Epidemiology and Biometry, University of Würzburg, Würzburg, Germany.

^22^ State Institute of Health I, Bavarian Health and Food Safety Authority, Erlangen, Germany

^23^ Institute for Medical Informatics, Biometry and Epidemiology, University Hospital of Essen, University of Duisburg-Essen, Germany

^24^ Department of Epidemiology, Helmholtz-Centre for Infection Research (HZI), Braunschweig, Germany

^25^ Molecular Epidemiology Research Group, Max Delbrück Center for Molecular Medicine (MDC) in the Helmholtz Association, Berlin, Germany

^26^ Biobank Technology Platform, Max Delbrück Center for Molecular Medicine (MDC) in the Helmholtz Association, Berlin, Germany

^27^ Charité - Universitätsmedizin Berlin, corporate member of Freie Universität Berlin and Humboldt-Universität zu Berlin, Berlin, Germany

**Corresponding author:**

Prof. Dr. med. Maximilian König, MSc

Klinik und Poliklinik für Innere Medizin D – Geriatrie

Universitätsmedizin Greifswald

D-17475 Greifswald

[maximilian.koenig@med.uni-greifswald.de](mailto:maximilian.koenig@med.uni-greifswald.de)

**Supplementary Table 1**. Frailty index items

| **Nr.** | **Variable name** | **Description** | **Coding** |
| --- | --- | --- | --- |
| 1 | rheuma_fi | Rheumatism (Arthritis/Polyarthritis) | 0 = no, 1 = yes |
| 2 | ckd_fi | Chronic Kidney Disease (CKD) | 0 = no, 1 = yes |
| 3 | asthma_fi | Asthma | 0 = no, 1 = yes |
| 4 | hyperlipidemia_fi | Elevated blood lipids (cholesterol or triglycerides) | 0 = no, 1 = yes |
| 5 | gout_fi | Gout | 0 = no, 1 = yes |
| 6 | sleep_fi | Sleep quality (Subjective sleep quality) | 1=0, 2=0.33, 3=0.66, 4=1 |
| 7 | thyroid_fi | Thyroid disease | 0 = no, 1 = yes |
| 8 | iadl8_fi | ... going to the doctor, pharmacy, physiotherapy, or other medical facilities (possibly using public transport)? | 0 = independent; 0.25; 0.5; 0.75; 1 =impaired |
| 9 | iadl7_fi | ... making phone calls? | 0 = independent; 0.25; 0.5; 0.75; 1 =impaired |
| 10 | iadl6_fi | ... receiving and entertaining visitors? | 0 = independent; 0.25; 0.5; 0.75; 1 =impaired |
| 11 | iadl5_fi | ... writing letters, filling out forms? | 0 = independent; 0.25; 0.5; 0.75; 1 =impaired |
| 12 | iadl4_fi | ... managing your financial affairs? | 0 = independent; 0.25; 0.5; 0.75; 1 =impaired |
| 13 | iadl3_fi | ... doing laundry, ironing? | 0 = independent; 0.25; 0.5; 0.75; 1 =impaired |
| 14 | iadl2_fi | ... shopping for food, clothing, household items, etc.? | 0 = independent; 0.25; 0.5; 0.75; 1 =impaired |
| 15 | iadl1_fi | ... cleaning your home? | 0 = independent; 0.25; 0.5; 0.75; 1 =impaired |
| 16 | satisfied_fi | Life satisfaction (“How satisfied are you currently, all in all, with your life?”) | 0, 0.2, …, 0.9, 1 |
| 17 | exhaustion_fi | Exhaustion (“During the past 7 days, to what extent have you suffered from unusual exhaustion and fatigue?”) | 0, 0.2, …, 0.9, 1 |
| 18 | subj_health_fi | Subjective health (“How would you describe your general state of health?”) | 0, 0.25, 0.5, 0.75, 1 |
| 19 | strength_fi | Loss of strength (“...climbing several flights of stairs”) | 0, 0.5, 1 |
| 20 | sad_fi | “...feeling discouraged and sad?” | 0, 0.5, 1 |
| 21 | fit_fi | Fitness (self-assessed) | 0, 0.1, 0.2, 0.3, 0.4, 0.5, 0.6, 0.7, 0.8, 0.9, 1 |
| 22 | fracture50_fi | Bone fracture after age 50 | 0 = no, 1 = yes |
| 23 | teeth_fi | Number of teeth | 0–9=0, 10–19=0.33, 20–26=0.66, 27+=1 |
| 24 | glaucoma_fi | Glaucoma | 0 = no, 1 = yes |
| 25 | macula_fi | Macular degeneration | 0 = no, 1 = yes |
| 26 | cataract_fi | Cataract | 0 = no, 1 = yes |
| 27 | mi_fi | Myocardial infarction | 0 = no, 1 = yes |
| 28 | cad_fi | Coronary heart disease (CHD) | 0 = no, 1 = yes |
| 29 | chf_fi | Congestive heart failure (CHF) | 0 = no, 1 = yes |
| 30 | arrhythmia_fi | Cardiac arrhythmia | 0 = no, 1 = yes |
| 31 | pad_fi | Peripheral arterial disease (PAD) | 0 = no, 1 = yes |
| 32 | dm_fi | Diabetes mellitus (any form) | 0 = no, 1 = yes |
| 33 | copd_fi | Chronic obstructive pulmonary disease (COPD) | 0 = no, 1 = yes |
| 34 | xirrhosis_fi | Liver cirrhosis | 0 = no, 1 = yes |
| 35 | polypharma_fi | Polypharmacy | „0–4“=0, „5–9“= 0.5, „≥10“ = 1 |
| 36 | backpain_fi | Back pain (“Back pain for 3 months or longer, almost daily”) | 0 = no, 1 = yes |
| 37 | osteoporosis_fi | Osteoporosis | 0 = no, 1 = yes |
| 38 | parkinson_fi | Parkinson’s disease | 0 = no, 1 = yes |
| 39 | stroke_fi | Stroke (Number of strokes) | 0 = no, 0.75 = yes, 1 = recurrent stroke |
| 40 | cancer_fi | Cancer (“Have you ever been diagnosed with cancer by a doctor?”) | 0 = no, 1 = yes |

***Notes and abbreviations*:** all information is self-reported, 0 generally indicates absence of deficit, 1 indicates presence of full deficit; these 10 candidate items were excluded during the selection process: Hearing impairment, Neuropathy, Dental prostheses, Hypertension, HIV, Diabetic foot, Gastric ulcer, Alcohol addiction, Vertigo, Osteoarthritis.

**Supplementary Table 2**. Regional variation in the prevalence of frailty, mean frailty index, and possible determinants

| **NAKO Study Center** | **Mean FI** | **Prevalence**  **of frailty (FI ≥ 0.25)** | **Age structure (61-64/65-69/70-75)** | **Mean net equivalence**  **Income** | **Weak social network** | **Subjective health^#^** | **Education^+^** | **Migration**  **Background** | **East/West** | **North/**  **South** | **Urbanization** | **Excluded*** |
| --- | --- | --- | --- | --- | --- | --- | --- | --- | --- | --- | --- | --- |
| Freiburg | 0.14±0.06 | 5.9 % | 44.2/48.1/7.8 % | 2586 € | 51.2 % | 2.86±0.70 | 5 (3;5) | 14.1 % | W | S | M | 18.8 % |
| Bremen | 0.14±0.06 | 6.0 % | 44.8/48.2/7.1 % | 2500 € | 49.8 % | 2.84±0.66 | 5 (3;5) | 10.3 % | W | N | U | 18.9 % |
| Münster | 0.14±0.06 | 6.0 % | 47.4/49.2/3.5 % | 2906 € | 47.8 % | 2.84±0.67 | 5 (3;5) | 8.6 % | W | N | U | 14.8 % |
| Hamburg | 0.14±0.06 | 6.3 % | 38.1/50.4/11.5 % | 2567 € | 54.9 % | 2.86±0.68 | 4 (3;5) | 14.3 % | W | N | U | 11.8 % |
| Hannover | 0.14±0.06 | 6.3 % | 42.9/48.7/8.4 % | 2620 € | 52.7 % | 2.88±0.66 | 5 (3;5) | 13.0 % | W | N | U | 25.5 % |
| Kiel | 0.15±0.07 | 7.1 % | 45.4/50.0/4.6 % | 2356 € | 51.1 % | 2.86±0.66 | 5 (3;5) | 9.9 % | W | N | M | 19.7 % |
| Berlin (South) | 0.15±0.06 | 7.2 % | 36.8/47.7/15.5 % | 2496 € | 61.7 % | 2.89±0.64 | 5 (3;5) | 15.4 % | W | C | U | 21.2 % |
| Mannheim | 0.15±0.06 | 7.4 % | 44.9/48.1/7.0 % | 2458 € | 51.6 % | 2.93±0.66 | 4 (3;5) | 18.8 % | W | S | U | 20.0 % |
| **Total** | **0.15±0.07** | **7.7 %** | **44.7/47,6/7.7 %** | **2306 €** | **51.6 %** | **2.92±0.65** | **5 (3;5)** | **11.8 %** | **-** | **-** | **-** | **22.3 %** |
| Neubrandenburg | 0.15±0.07 | 7.7 % | 51.3/44.9/3.8 % | 1819 € | 48.7 % | 3.01±0.60 | 5 (3;5) | 7.3 % | E | N | S/R | 39.9 % |
| Augsburg | 0.15±0.07 | 7.8 % | 45.2/46.8/8.0 % | 2260 € | 46.6 % | 2.90±0.68 | 3 (3;5) | 16.8 % | W | S | M | 23.9 % |
| Halle | 0.15±0.07 | 8.5 % | 43.5/48.5/8.0 % | 1852 € | 50.9 % | 3.01±0.57 | 5 (3;5) | 6.2 % | E | C | M | 26.0 % |
| Leipzig | 0.15±0.07 | 8.6 % | 45.5/44.7/9.7 % | 1711 € | 50.2 % | 3.06±0.59 | 5 (3;5) | 6.6 % | E | C | U | 10.4 % |
| Berlin (North) | 0.15±0.07 | 8.7 % | 47.9/48.2/3.9 % | 2049 € | 53.7 % | 2.96±0.59 | 5 (3;5) | 6.0 % | E | C | U | 13.5 % |
| Düsseldorf | 0.15±0.07 | 8.9 % | 43.3/49.2/7.5 % | 2695 € | 58.9 % | 2.88±0.67 | 5 (3;5) | 18.8 % | W | C | U | 30.1 % |
| Saarbrücken | 0.15±0.07 | 8.9 % | 45.8/50.7/3.51 % | 2338 € | 44.8 % | 2.91±0.65 | 4 (3;5) | 9.7 % | W | S | M | 25.7 % |
| Regensburg | 0.16±0.07 | 8.9 % | 43.8/45.2/11.1 % | 2307 € | 43.7 % | 2.96±0.65 | 3 (3;5) | 12.0 % | W | S | M | 24.5 % |
| Berlin (Mitte) | 0.16±0.07 | 9.4 % | 43.1/45.5/11.4 % | 2204 € | 64.7 % | 2.95±0.67 | 5 (3;5) | 12.4 % | E | C | U | 15.9 % |
| Essen | 0.16±0.07 | 10.3 % | 46.4/47.1/6.5 % | 2424 € | 51.0 % | 3.00±0.67 | 4 (3;5) | 13.9 % | W | C | U | 17.0 % |

***Notes and abbreviations:*** Data are presented as mean w/ and w/o (±SD), percentages, median (25^th^;7^th^ percentile); SES = Socio-Economic Status (European Socio-economic Classification), Weak social network = Social Network Index Level I or II, Urbanization: U=urban, S=suburban, R=rural, M=mixture of all three categories according to Wolf et al. [28], C=Central, N= North, S= South, W= West, E = East, FI = Frailty Index, ^#^Subjective Health: 1=excellent, 2=very good, 3= good, 4 = fair, 5 = bad; ^+^Education: ISCED-97 (International Standard Classification of Education, 1997 version) defines levels of education from 0 to 6; *this proportion of participants was excluded from the analyses because the Frailty Index (FI) could not be computed when more than 20% of FI items were missing

**Supplementary Figure 1. Study flow chart**


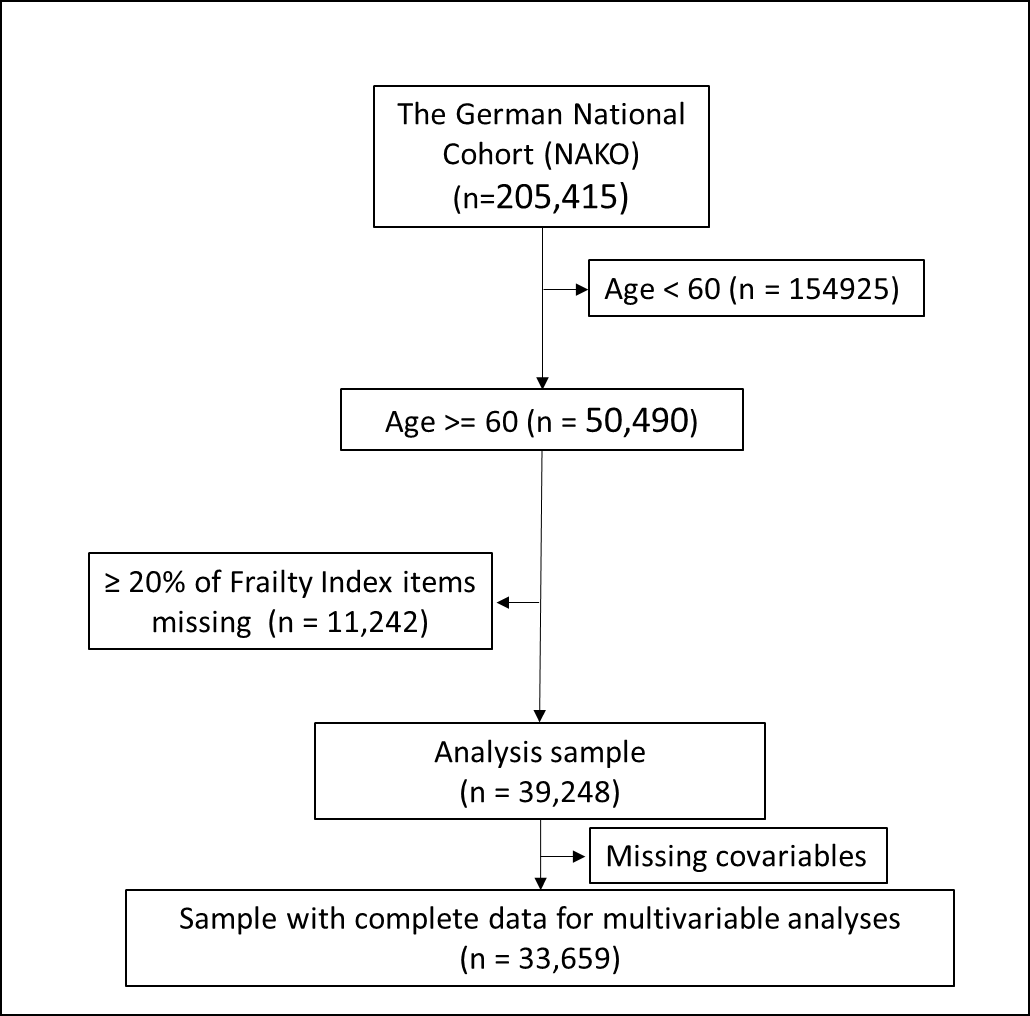


**Supplementary Table 3.** Frailty distribution by urbanization

| **Frailty category** | **Urban**  (n =22,499) | **Mixture**  (n=13,359) | **Suburban/Rural**  (n=3,390) | **Total**  (n=39,248) |
| --- | --- | --- | --- | --- |
| **Robust/Prefrail** | 20,772 (92.3 %) | 12,313 (92.2 %) | 3,130 (92.3 %) | 36,215 (92.3 %) |
| **Frail** | 1,727 (7.7 %) | 1,046 (7.8 %) | 260 (7.7 %) | 3,033 (7.7%) |

**Notes:** Data are presented as numbers(percentages), Pearson chi²(2) = 0.297, p = 0.862

**Supplementary Table 4.** Prevalence of frailty across the study sites (design-weighted)

| **Frailty category** | **Augsburg** | **Regensburg** | **Mannheim** | **Freiburg** | **Saarbrücken** | **Essen** | **Münster** | **Düsseldorf** | **Halle** | **Leipzig** | **Berlin Nord** | **Berlin Mitte** | **Berlin Süd** | **Hannover** | **Hamburg** | **Bremen** | **Kiel** | **Neubrandenburg** | **Total** |
| --- | --- | --- | --- | --- | --- | --- | --- | --- | --- | --- | --- | --- | --- | --- | --- | --- | --- | --- | --- |
| **Robust, %** | 56.87 | 51.56 | 59.14 | 61.16 | 54.77 | 52.10 | 63.00 | 56.81 | 56.38 | 54.89 | 56.43 | 54.34 | 58.16 | 63.40 | 61.47 | 62.26 | 61.57 | 60.94 | 58.10 |
| **Prefrail, %** | 35.57 | 39.20 | 33.42 | 33.06 | 36.84 | 37.58 | 31.55 | 34.17 | 35.07 | 36.52 | 34.88 | 36.23 | 34.49 | 30.22 | 32.21 | 31.68 | 31.49 | 31.43 | 34.13 |
| **Frail, %** | 7.56 | 9.24 | 7.44 | 5.79 | 8.39 | 10.32 | 5.45 | 9.02 | 8.55 | 8.59 | 8.69 | 9.44 | 7.35 | 6.38 | 6.31 | 6.06 | 6.94 | 7.63 | 7.78 |
| **Total, %** | 100 | 100 | 100 | 100 | 100 | 100 | 100 | 100 | 100 | 100 | 100 | 100 | 100 | 100 | 100 | 100 | 100 | 100 | 100 |

**Supplementary Table 5.** Individual-level associations of frailty (multivariable logistic regression, design-weighted), n = 33,659.

|  | **OR (95% CI)** | **p-value** |
| --- | --- | --- |
| **Male Sex** | 1 (Ref.) | — |
| **Female Sex** | 0.99 (0.88–1.12) | 0.867 |
| **Age, years** | 1.04 (1.02–1.06) | <0.001 |
| **Income (ordinal)** | 0.73 (0.71–0.76) | <0.001 |
| **Education (ordinal)** | 0.87 (0.82–0.92) | <0.001 |
| **w/ immigr. background** | 1.00 (Ref.) |  |
| **w/o immigr. background** | 1.06 (0.92–1.22) | 0.419 |
| **Social network index (ordinal)** | 0.79 (0.72–0.86) | <0.001 |
| **Study site** |  |  |
| Augsburg | 1.00 (Ref.) |  |
| Regensburg | 1.41 (1.39–1.43) | <0.001 |
| Mannheim | 1.13 (1.11–1.15) | <0.001 |
| Freiburg | 0.88 (0.86–0.89) | <0.001 |
| Saarbrücken | 1.31 (1.29–1.33) | <0.001 |
| Essen | 1.53 (1.52–1.55) | <0.001 |
| Münster | 0.97 (0.94–0.99) | 0.032 |
| Düsseldorf | 1.47 (1.43–1.50) | <0.001 |
| Halle | 1.05 (1.00–1.10) | 0.038 |
| Leipzig | 1.01 (0.97–1.06) | 0.493 |
| Berlin Nord | 1.27 (1.22–1.32) | <0.001 |
| Berlin Mitte | 1.23 (1.19–1.28) | <0.001 |
| Berlin Süd | 1.10 (1.05–1.14) | <0.001 |
| Hannover | 1.05 (1.03–1.08) | 0.001 |
| Hamburg | 0.95 (0.92–0.97) | <0.001 |
| Bremen | 0.90 (0.88–0.92) | <0.001 |
| Kiel | 1.03 (1.02–1.05) | 0.001 |
| Neubrandenburg | 0.94 (0.90–0.99) | 0.014 |

***Notes and abbreviations:*** Odds ratios (OR) and 95% confidence intervals (CI), w/ = with, w/o = without

**Supplementary Table 6.** Comparison of Recruitment (n = 50490), Analysis (n = 39248)

and Complete Covariable Samples (n = 33659).

|  | **Recruiment**  **(n = 50490)** | **Analysis**  **(n = 39248)** | **Complete Covariable Sample**  **(n = 33659)** |
| --- | --- | --- | --- |
| **Age** | 65.3±2.9 | 65.2±2.8 | 65.1±2.8 |
| **Males sex** | 25261 (50.3) | 20506 (52.3) | 18058 (53.6) |
| **Migration status** | 7,254 (14.4) | 4,643 (11.8) | 3,808 (11.3) |
| **Income** | 2168 ± 1481 | 2306 ± 1531 | 2344 ± 1558 |
| **Sample composition, %** |  |  |  |
| - Augsburg | 10.13 | 9.92 | 9.48 |
| - Regensburg | 4.94 | 4.79 | 4.74 |
| - Mannheim | 4.85 | 5.00 | 5.01 |
| - Freiburg | 4.84 | 5.06 | 5.24 |
| - Saarbrücken | 5.01 | 4.79 | 4.15 |
| - Essen | 4.85 | 5.17 | 5.34 |
| - Münster | 4.82 | 5.29 | 5.33 |
| - Düsseldorf | 4.36 | 3.92 | 3.89 |
| - Halle | 5.04 | 4.80 | 4.88 |
| - Leipzig | 5.22 | 6.02 | 6.46 |
| - Berlin Nord | 4.88 | 5.43 | 5.57 |
| - Berlin Mitte | 5.15 | 5.57 | 5.75 |
| - Berlin Süd | 5.02 | 5.09 | 5.20 |
| - Hannover | 5.23 | 5.02 | 4.96 |
| - Hamburg | 4.89 | 5.55 | 5.62 |
| - Bremen | 5.04 | 5.26 | 5.37 |
| - Kiel | 4.52 | 4.67 | 4.74 |
| - Neubrandenburg | 11.18 | 8,64 | 8,27 |

***Notes:*** Values are presented as mean ± standard deviation for continuous variables and as absolute numbers (percentages)

for categorical variables. Percentages refer to the respective column sample size. Sample composition by study center

is shown as relative frequencies (%). Percentages for study centers do not sum exactly to 100% due to rounding.

**Supplementary Table 7.** Absolute sample sizes and frequencies of robust, prefrail, and frail, *total and by sex*

**Robust prefrail frail total**

**MEN**

61-64 years 5,754 2,564 543 8,861

64.94 % 28.94 % 6.13 % 100.00 %

65-69 years 5,952 3,196 779 9,927

59.96 % 32.20 % 7.85 % 100.00 %

70-75 years 888 668 162 1,718

51.69 % 38.88 % 9.43 % 100.00 %

**WOMEN**

61-64 years 5,096 2,923 667 8,686

58.67 % 33.65 % 7.68 % 100.00 %

65-69 years 4,585 3,426 726 8,737

52.48 % 39.21 % 8.31 % 100.00 %

70-75 years 564 599 156 1,319

42.76 % 45.41 % 11.83 % 100.00 %

Total 22,839 13,376 3,033 39,248

58.19 % 34.08 % 7.73 % 100.00 %
